# Supplementary figures and images for: CMV seropositivity is a potential novel risk factor for severe COVID-19 in non-geriatric patients
Source: PLoS One. 2022 May 25;17(5):e0268530. doi: 10.1371/journal.pone.0268530 (PMC9132318; doi:10.1371/journal.pone.0268530)

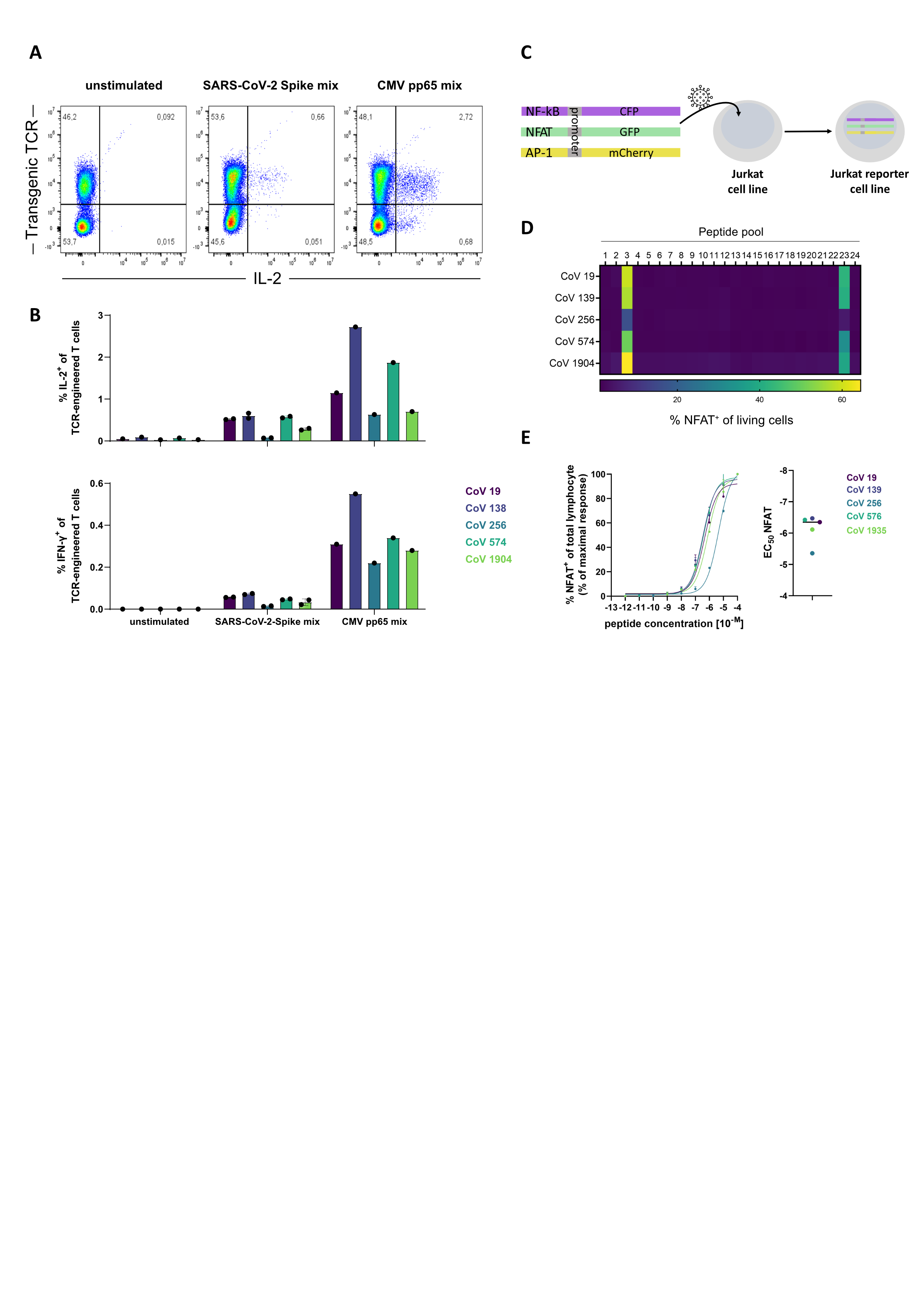

Supplement: S1 Fig — A-B) TCRs were isolated from an ICU patient and engineered into PBMCs from healthy donors via CRISPR/Cas9-mediated knock-in. Engineered T cells were co-cultured with autologous PBMCs previously pulsed with 1 μg/ml Peptivator S mix or CMV pp65 mix for 4 h at 37°C. Shown are representative raw data (A) and quantification (B) of IL-2 and IFN-γ production. C) Schematic depiction of the J-TPR system. Briefly, fluorescent protein genes were engineered downstream to TCR-triggered transcription factors. T cell activation can therefore be monitored by activation of the reporter genes. D) Overlapping peptides are generated from the CMV pp65 antigen and pooled into 24 subpools. Depicted is a summary heat map showing NFAT responses of TCR-engineered J-TPR cells after 18 h of co-culture with autologous CD40 activated B cells pulsed with 1 μg/ml of each individual subpool. The epitope AGILARNLVPMVAT is the one shared among pool 3 and 24. E) TCR-engineered J-TPR cells were co-cultured with autologous CD40 activated B cells pulsed with different AGILARNLVPMVAT peptide concentrations for 18 h at 37°C. Shown are NFAT reporter EC50 curves (left) and quantification (right). (TIFF) [file pone.0268530.s001.tiff]

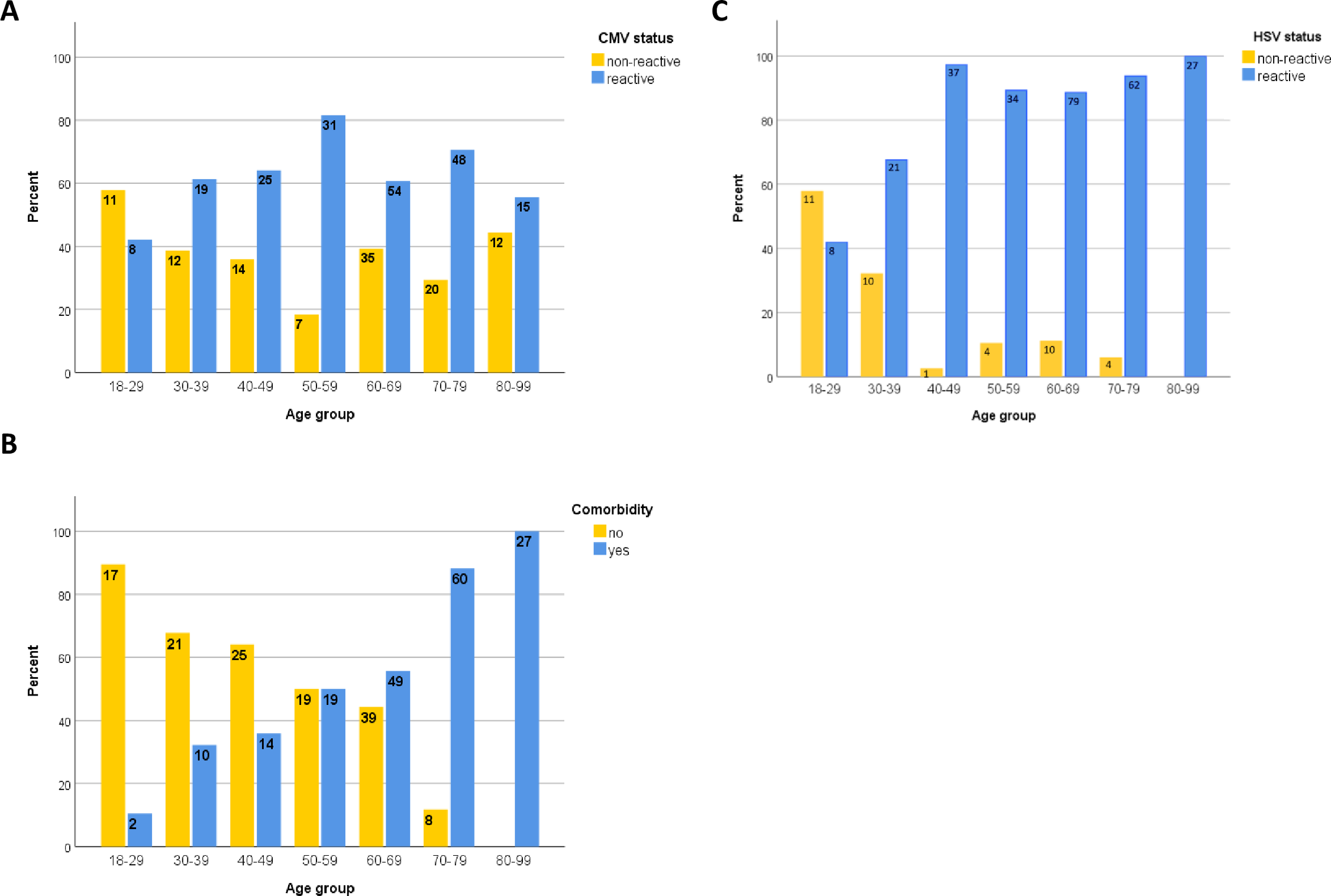

Supplement: S2 Fig — Bar graphs showing the percentage of individuals enrolled in this study positive or negative for CMV (A) and HSV serostatus (B), and with or without comorbidities (C). Numbers within the bars indicate absolute numbers of individuals. (TIF) [file pone.0268530.s002.tif]

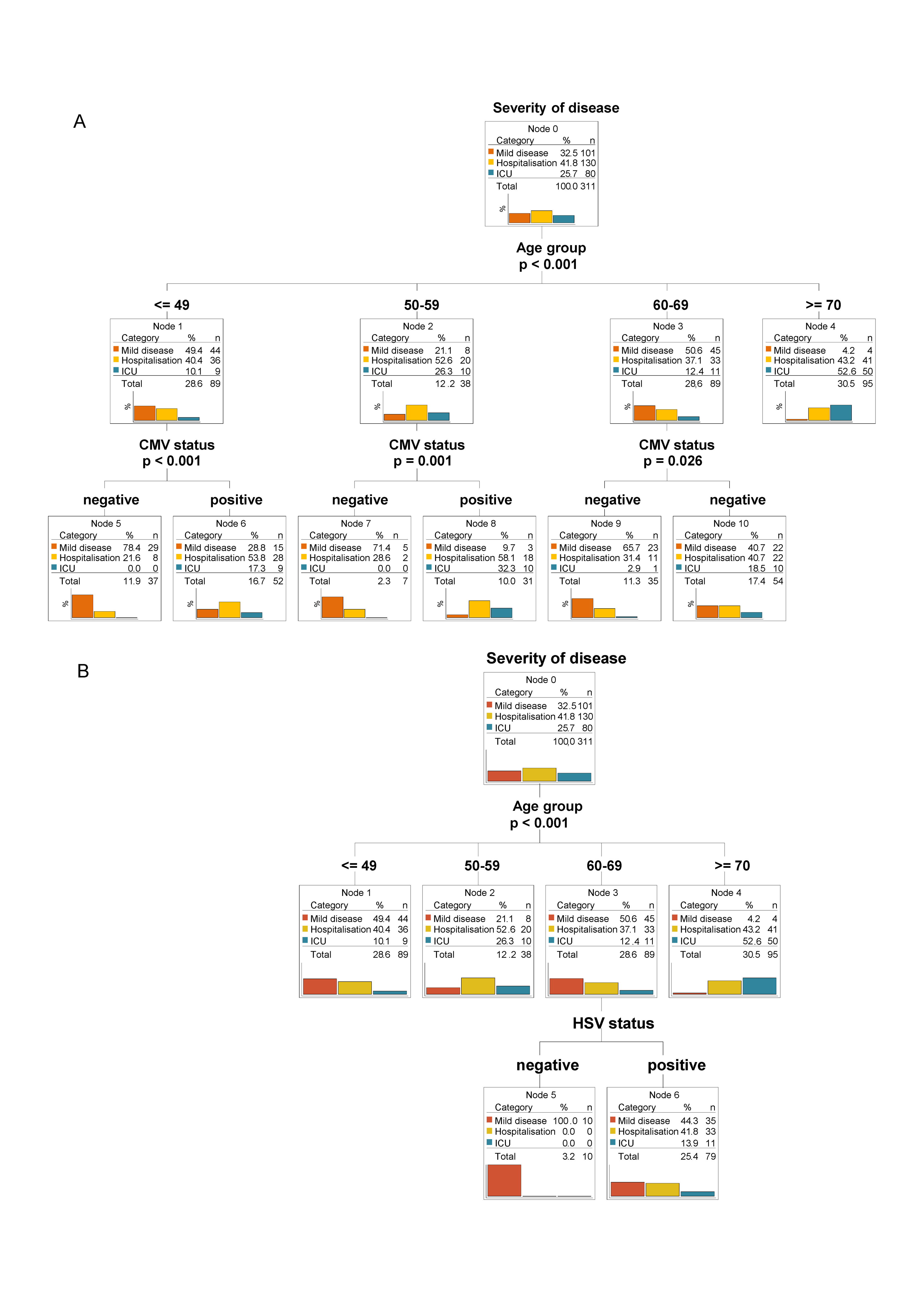

Supplement: S3 Fig — Classification tree model (CHAID) using age and either CMV serostatus or HSV serostatus as predictors of severity of disease. Bar plots represent percentages. Percentages for categories (mild disease, hospitalization and ICU) are calculated within the node. Percentages for the totals are calculated using the entire dataset. (TIF) [file pone.0268530.s003.tif]
